# Measuring potential interest in a postpartum contraceptive vaginal ring among breastfeeding women in India

**DOI:** 10.1371/journal.pgph.0000804

**Published:** 2022-07-27

**Authors:** Lorna Begg, Jim Sailer, Avishek Hazra, Malabika Roy, Ruth Merkatz, Lisa Haddad, Rebecca Brodsky, John Bongaarts

**Affiliations:** 1 Population Council, New York, NY, United States of America; 2 Population Council, New Delhi, India; 3 Indian Council of Medical Research, New Delhi, India; Tsinghua University, CHINA

## Abstract

Access to safe and effective contraception for postpartum women is an important priority in India, where the unmet need for postpartum contraception is high. In this paper, we estimate the potential market size in India for the progesterone vaginal ring (PVR), a novel user-controlled contraceptive method that offers additional contraceptive choice for lactating women. We integrated results of a one-year phase-3 multicenter clinical trial for the PVR conducted in India with an analysis of the National Family Health Survey (2015–16) and 2019 United Nations Population Division data to generate three estimates of potential market size for the PVR among postpartum breastfeeding women in India. We estimate the potential market size for the PVR ranges from a low estimate of 543,262 women to a high estimate of 1.3 million women, with a separate intermediate estimate of 737,460 women. Our analysis indicates the PVR could play an important role in decreasing unmet need among postpartum women in India, thereby reducing risks to mothers and children associated with short birth intervals, helping to prevent unintended pregnancies, and helping to address access-related issues heightened by the COVID-19 pandemic.

## Introduction

Providing safe and effective contraceptives is essential to achieving global commitments to improve the status of women and girls. Yet an estimated 218 million women ages 15–49 years living in low- and middle-income countries (LMICs) have an unmet need for contraception, meaning they want to avoid pregnancy but are not using a modern method [1]. Unmet need is particularly high among postpartum women, ranging between 48.5% and 62% across LMIC regions and is highest in South and South East Asia [2, 3]. The COVID-19 pandemic has only amplified the sexual and reproductive health needs of women and girls. Lockdowns and travel restrictions implemented around the world to slow the transmission of COVID-19 have often included shutting down sexual and reproductive healthcare facilities, which are not universally classified as essential, thereby exacerbating access-related barriers to contraception [4]. Lockdowns have had devastating effects on sexual and reproductive healthcare access in India. In 2020–21, as compared to 2019–20, sterilization decreased by 22% and intrauterine copper devices (IUCDs) and progestin injectable use decreased by 6.3% and 3.7% respectively [5].

The disruptions to family planning services in India have a variety of repercussions, including restricting women’s ability to extend birth intervals. The World Health Organization (WHO) recommends birth intervals of 2–3 years to reduce the risk of adverse maternal and child health outcomes [6]. The vast majority of women with an unmet need postpartum want to avoid subsequent pregnancies for at least 2 years after birth [2, 3]. By reducing unmet need and lengthening birth intervals, the risks of preterm birth, low birth weight, and maternal and infant mortality can be reduced [2, 7]. Exclusive or near-exclusive breastfeeding, e.g., the lactational amenorrhea method (LAM), delays the resumption of normal ovarian cycles and promotes postpartum amenorrhea, resulting in longer birth intervals. It is 98% effective as pregnancy prevention, but only for the first 6 months postpartum [8]. Breastfeeding also provides essential infant nutrition and is one of the most effective ways to ensure child health and survival, preventing 20% of newborn deaths [9]. Accordingly, the WHO and the United Nations Children’s Emergency Fund recommend infants be exclusively breastfed for at least the first 6 months of life [10]. Despite the benefits of exclusive breastfeeding, however, 36% of children in India are not exclusively breastfed, with inadequate breastfeeding contributing to 100,000 preventable child deaths and 37 million cases of diarrhea and pneumonia annually [11, 12].

The contraceptive options considered safe for breastfeeding women available in India through the national family planning program are Centchroman weekly oral pills, male condoms, the copper-intrauterine contraceptive device, progestin-only injectables, and tubal ligation [13]. The provision of these family planning methods, however, reduced during the COVID-19 pandemic [5]. Irrespective of COVID-19 access-related issues, each of these methods has limitations. Centchroman weekly oral pills must be taken twice a week starting on the first day of menstruation, then 3 days after, for the first 3 months followed by once a week thereafter [14]. This adherence requirement can be challenging, especially for new mothers. While condoms are available, couples often have difficulty with consistent use [15]. Long-acting reversible methods, like injections or IUCDs, offer effective pregnancy prevention but require trained provider insertion which can be a barrier in LMICs where supply-side deficits, including lack of trained providers, are common [16]. Although the method mix has expanded, tubal ligation dominates the method mix in India, with about 57% of all contraceptive users relying on this permanent method [12]. Although tubal ligation is a postpartum option, it may not be appropriate for women who are uncertain about future childbearing.

Long-acting methods and tubal ligation require trained provider interaction, which have added complication in the era of COVID-19 when public health clinics have been forced to close or be repurposed for COVID-19-related care [4]. New user-controlled, reversible methods that do not require specifically trained provider interaction could help alleviate contraceptive access barriers. One such method is the progesterone vaginal ring (PVR), a 3-month intravaginal ring releasing ~10 mg of progesterone per day that can be inserted and removed by the user starting 4 weeks after birth, used continuously, and replaced every 3 months while breastfeeding is continued. The slow release of progesterone prolongs the duration of lactational amenorrhea, suppresses ovulation, and does not affect the quality of breastmilk [17–23]. In clinical trials, the PVR was found to be as effective for contraception as the IUCD [8, 17, 22]. The effectiveness of the PVR requires the user to breastfeed her child at least four times a day, with fewer feeds reducing effectiveness. Hence, in places like India where breastfeeding is an accepted practice but exclusive breastfeeding is limited, the PVR could provide highly effective contraception while promoting prolonged breastfeeding and supporting infant growth and well-being [8, 22].

The PVR is registered in ten Latin American countries, Nigeria, and Senegal and is included on the WHO Essential Medicines List [24]. Its availability, however, is impeded by lack of clarity on the potential market size, limiting product promotion among pharmaceutical companies. In this paper, we estimate the potential market size for the PVR in India. The goal is to create a point-in-time “idealized” market estimate to better understand the product’s potential. Market estimates can never be precise as they rely on a variety of assumptions. Nevertheless, they remain valuable and sought-after tools to generate support among stakeholders, including commercial partners, donors, policy makers, and government entities. With this market size estimate, we hope to generate interest in the PVR and ensure wider availability of the PVR to address postpartum unmet need, including during this challenging time of the COVID-19 pandemic.

## Methods

Our PVR market size estimates are based on three data sources. First are the findings from the clinical trial of the PVR conducted at 20 medical colleges across 13 states in India by the Indian Council of Medical Research and the Population Council (Trial registration number: CTRI/2011/07/001874) [8]. The objectives of this trial were to assess safety and efficacy, continuation rates, duration of lactational amenorrhea, breastfeeding performance, and infant growth and health associated with the PVR among lactating women seeking postpartum contraception. The PVR study was approved by the ethics committees of the Indian Council of Medical Research, the 20 participating centers, and the Population Council’s Institutional Review Board [8]. Trained personnel counselled lactating women 6–9 weeks postpartum on contraceptive options during their visits to family planning and well-baby clinics. Women who expressed willingness to accept a contraceptive method and participate in the clinical trial were screened for eligibility and enrollment [8]. Participants were healthy women ages 20–35 years, 6–9 weeks postpartum, exclusively breastfeeding, willing to continue breastfeeding at least four times daily for one year, had not used contraceptives since delivery, were at risk for pregnancy, and were willing to come for follow-up visits. Women who selected the PVR were trained on self-insertion and removal and were instructed to keep daily diaries to track bleeding, removals, and expulsions.

Second are data from the 2015–16 Indian National Family Health Survey (NFHS-4), equivalent to the Demographic Health Survey (DHS), which provide estimates on the proportion of postpartum women who are breastfeeding and who have unmet need for contraception. At the time of this analysis, the 2015–16 NFHS-4 results were the most recent results available. Although NFHS-5 has now been released, the clinical trial’s data collection period and 2015–16 NFHS data time frame are similar and, therefore, we have kept this dataset for our estimates. The NFHS is nationally representative with a sample of 628,892 residential households [25]. Using the statistical program STATA, tabulations were extracted from the NFHS-4 2015–16 data file, yielding distributions of women by current contraceptive method, unmet need, breastfeeding status, and length of time since last birth.

Third are data from the 2019 United Nations Population Division (UNPD), which provide an estimate of the number of mothers in India with children aged under 1 year in 2020 [26].

Based on uptake of the PVR during the clinical trial, we calculated three estimates ranging from conservative to ambitious to indicate a potential market size for the PVR.

### Ethics statement

No patients were involved in this market size analysis.

## Results

Using NFHS-4 and UNPD data, we estimate there are 24.1 million women in India with a child under age 1 year in 2020. Among these women, 94% are breastfeeding. Similar to other studies, we utilized the DHS variable ‘predominant breastfeeding,’ which is defined as women who provide their infants with breastmilk as their main source of nourishment and only supplement with water or other non-milk liquids [27]. In this paper, the term ‘predominant breastfeeding’ includes women who exclusively breastfeed and those who supplement only with water or non-milk liquids. Using this variable, we calculated 51% (12.3 million) of women with a child under age 1 year in India are predominantly breastfeeding and are potential candidates for the PVR (see **Table 1**). Nineteen percent of these women are already using traditional or modern contraception, 55% have no interest in contraception, and 26% has an unmet need for contraception. The estimated number of predominantly breastfeeding women with an unmet need for contraception is 3.2 million.

**Table 1:** Number of women by breastfeeding and contraceptive status, India 2020.

		Million	Percent
**A. Breastfeeding status of postpartum women**			
	Women with child under age 1 year	24.1	100%
	Women breastfeeding	22.7	94%
	** Women predominantly breastfeeding**	**12.3**	**51%**
**B. Contraceptive status of postpartum women predominantly breastfeeding (12.3 million)**			
	Using any contraceptive method	2.3	19%
	No interest in contraception	6.8	55%
	** Unmet need for contraception**	**3.2**	**26%**

**Fig 1:**
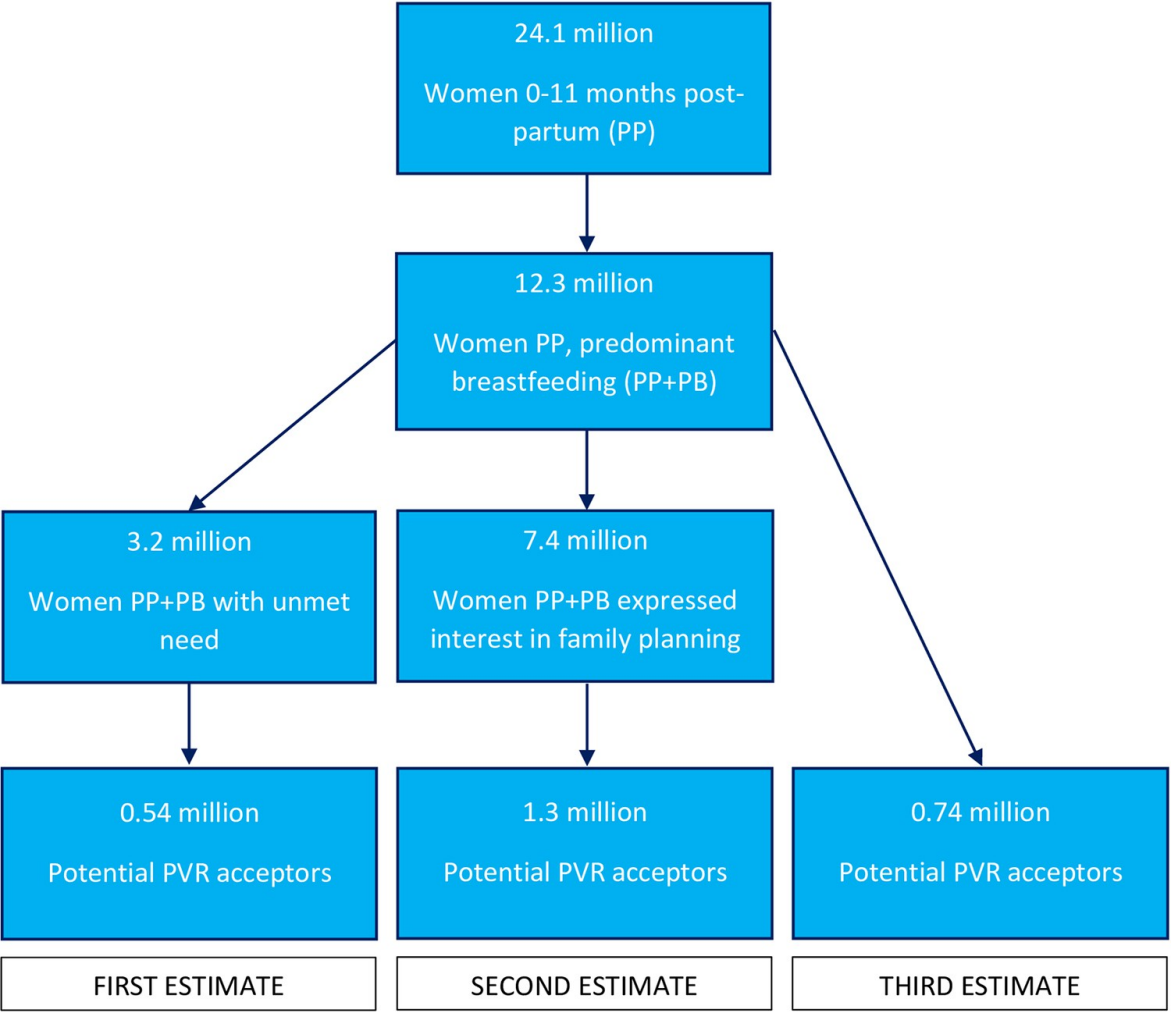
PVR market size estimates.

There are limited available data to help us estimate how many predominantly breastfeeding women may be interested in the PVR. As such, we relied on the results of the PVR phase III clinical trial to estimate method acceptance among postpartum women in India [8]. In the trial, of the 14,835 lactating postpartum women counselled, 60% (8,978) expressed willingness to accept a contraceptive method [8]. Of those, 17% (1,528) were willing to accept PVR; 5% (459) were found to be eligible for the clinical trial and the remaining 12% (1,069) were ineligible for the trial. Applying the 17% to the 3.2 million women with an unmet need yields our first market estimate of 543,262 (see left column of **Fig 1**).

For our second estimate, we relied on the expressed interest in any family planning method as observed in the PVR clinical trial. In the trial, 60% of lactating women counselled expressed interest in any family planning method. Therefore, we estimate the number of postpartum predominantly breastfeeding women who may be interested in any family planning method is 7.4 million (60% of the 12.3 million women who predominantly breastfeed). In the trial, 17% of these women were willing to use the PVR. Applying this percentage to the estimated number of women interested in any method (17% of 7.4 million) yields our second market estimate of 1,253,682 women (see middle column in **Fig 1**).

Our third estimate relies on historical data of contraceptive prevalence trends in LMICs. In the early decades of family planning programs, governments started new programs by providing a limited set of contraceptive methods. Over time, new methods were added, thus giving women and men wider choice. When the method mix is expanded, overall contraceptive prevalence increases [28]. A review of the evidence on past program expansions concluded that adding a new method can be expected to increase contraceptive prevalence by about 6% net of the effect of socioeconomic development [28]. If we assume 6% adoption of the PVR among the 12.3 million postpartum women predominantly breastfeeding, then we obtain a third estimate of 737,460 (6% of 12.3 million) women (see right column of **Fig 1**).

The three estimates calculated above suggest that the potential market size for the PVR ranges from 543,262 women to 1,253,682 women annually, with a separate intermediate estimate of 737,460 women.

## Discussion

Based on our calculations, roughly 24 million women in India gave birth in 2020, 48% of whom were adolescent girls and young women (15–24 years). The most used method during the postpartum period was permanent contraception, defined in the DHS as female sterilization, with an estimated 1.7 million postpartum women sterilized annually. Female sterilization is performed at young ages, with 43% of currently sterilized women sterilized before the age of 25 years [29]. Since young women are more likely to express permanent contraception regret, expanding choice for highly effective, reversible contraception safe to use during breastfeeding could allow couples to delay female sterilization and avert permanent contraception regret [30, 31].

Although female sterilization dominates the method mix in India, there have been increases in the adoption of oral contraceptive pills and condoms, particularly among young users, less educated users, and users from poorer households, coupled with a decline in traditional method use over the past two decades [32]. This shift can likely be credited to government initiatives to supply condoms and oral contraceptive pills at minimal cost at the doorstep by Accredited Social Health Activists (community health workers) [32]. In India, the proportion of deliveries occurring in healthcare facilities rather than at home has increased from 38.7% in 2005–2006 to 88.6% in 2019–21 [33], largely as a result of the “Janani Suraksha Yojana”—a conditional cash transfer scheme to promote institutional deliveries and reduce maternal deaths [34]. Given women’s increased exposure to family planning at healthcare facilities, the immediate postpartum period presents an opportunity to counsel women on contraceptive options including the PVR. A woman could be given the PVR prior to discharge along with a package insert detailing use instructions to start at four weeks after birth or at her postpartum visit for continuous use up to 3 months. The PVR would not require significant healthcare infrastructure for insertion and would help reduce travel costs associated with postpartum follow-up. The roll-out of the PVR would especially benefit from the engagement of Accredited Social Health Activists to reach women at the doorstep.

Most Indian women consult numerous family members including their partner, mother, mother-in-law, or sister while deciding on the use of contraception [35]. In the clinical trial for the PVR, some of the most commonly cited reasons among the 40% of women who did not express interest in any family planning method was a need to discuss with family members. Given the influence of family members on a woman’s decision for contraception, educational campaigns that include husbands and mothers-in-law that focus on the health benefit for women and infants will be critical to the roll-out of the PVR.

Although the PVR has many benefits, there are several requirements for successful use. First, to maintain ovulation suppression with the PVR, women must breastfeed at least four times each day, a target that may be difficult to achieve if women are unable to breastfeed exclusively or frequently. Second, the PVR must be replaced every 3 months, a requirement that may present practical challenges in low resource or rural settings, especially during COVID-19 lockdowns or mobility restrictions. During the clinical trial, ring expulsions occurred, often due to the squatting position used for toileting or during housework. Counseling on the risks of expulsions and practices to prevent expulsions are essential to ensure correct use of the PVR, especially in regions where squatting toilets are common. Women need to be confident about placing the ring correctly and need to be provided with strategies to avoid expulsions during these situations. Finally, the PVR is effective for a three-month duration and obtaining new vaginal rings every three months may be relatively expensive or logistically difficult.

In response to challenges of PVR use, the Population Council is developing a new longer-acting contraceptive vaginal ring for postpartum women containing segesterone acetate. A segesterone acetate vaginal ring would be safe to use during breastfeeding, would not require a fixed number of daily feeds, and would have efficacy for 12-months continuous use [36]. This longer-acting vaginal ring could alleviate service delivery issues associated with a 3-month ring, especially given lack of follow-up and access inequalities to postnatal care [30]. Segesterone acetate blocks ovulation in non-lactating women and could be used after the postpartum period. Although a segesterone acetate contraceptive vaginal ring has not yet been clinically tested in lactating women, there have been three clinical studies performed in lactating women of segesterone acetate implants, all of which indicated no safety signals related to growth and development of breastfed infants [36–39]. A segesterone acetate one-year vaginal ring would be a beneficial and cost-effective option in the era of COVID-19, as it would not require provider interaction or frequent clinic access.

### Limitations

The PVR market analysis described in this paper did not address issues related to supply chain networks, regulatory requirements, procurement, and other infrastructure needs necessary to achieve this level of uptake. If regulatory approval is achieved, effective counseling and informational campaigns will be essential to spread awareness of the method and enable uptake. If the Indian government-sponsored family planning program incorporates this method at a low-cost to users, this will increase accessibility.

There is a high level of uncertainty in market size assessments, as accurate and comprehensive data are lacking to estimate uptake, especially in LMIC markets. Hence, we used the available clinical uptake data for the PVR among Indian women as the most reliable indicator of women’s preferences. Using clinical trial results to measure wider market uptake has limitations, as it assumes trial participants are representative of women in India. The potential interest in the PVR generated from the clinical trial data may be skewed by social desirability bias as the trial was centered around the PVR and counseling could have placed emphasis on the ring.

Given high rates of unmet need and newborn deaths coupled with low rates of reversible contraceptive use in many global regions including Southeast Asia, further investigation is needed to better understand the larger potential market for the PVR. Further analysis will also be needed to generate a multi-year uptake estimate.

## Conclusion

There continues to be high unmet need for postpartum contraception in India which can only increase as COVID-19 continues to disrupt sexual and reproductive healthcare services. A user-controlled progesterone vaginal ring could reduce the risk for unintended pregnancy, increase contraceptive choice, and help to address access-related issues heightened by the COVID-19 pandemic. The introduction of a new, reversible contraceptive option in India could help provide a more balanced method mix and may alleviate the access-related issues exacerbated by COVID-19. There will be typical barriers to uptake of family planning methods including cost, provider knowledge, counseling requirements, method availability, as well as barriers unique to India, including high rates of female sterilization and influence of family members on contraceptive decisions. However, if enough interest in the PVR is generated to help address these barriers, this product could play a role for postpartum breastfeeding women seeking contraception.
